# Thoracoscopic pneumonectomy in patient with unilateral absence of the left pulmonary artery accompanied by the left lung cancer

**DOI:** 10.1093/icvts/ivab364

**Published:** 2022-01-17

**Authors:** Pavel V Kononets, Parvin I Akhmedov, Konstantin Y Topol

**Affiliations:** 1 Department of Thoracic Surgery, N.N. Blokhin NMRC of Oncology, Moscow, Russian Federation; 2 Department of Diagnostic Radiology, N.N. Blokhin NMRC of Oncology, Moscow, Russian Federation

**Keywords:** Absence of pulmonary artery, Thoracoscopy, Lung adenocarcinoma

## Abstract

Unilateral absence of the pulmonary artery is a rare congenital cardiovascular anomaly. Unilateral absence of the pulmonary artery is often accompanied by cardiovascular disorders but also can occur in an isolated manner. We present a case of female patient, in which the absence of the left pulmonary artery was revealed and the left lower lobe adenocarcinoma was diagnosed.

## INTRODUCTION

Unilateral absence of the pulmonary artery (UAPA), also known as unilateral pulmonary artery agenesis, was first described by Frantzel in 1868 [1]. With an estimated prevalence of 1 in 200 000 individuals [2], UAPA is a rare congenital cardiovascular anomaly that is caused by the involution of the proximal sixth aortic arch and persistence of the connection of the intrapulmonary pulmonary artery to the distal sixth aortic arch [3]. UAPA is often accompanied by cardiovascular disorders, such as tetralogy of Fallot, cardiac septal defects, truncus arteriosus or aortic arch anomalies, but also can occur in an isolated manner. Symptoms of isolated UAPA include dyspnoea, respiratory infections, haemoptysis and pulmonary hypertension, but can remain asymptomatic [4]. The incidence of the lung cancer in patients with UAPA is very rare and surgical experience is limited. We report a clinical case of a female with isolated left UAPA and right aortic arch, who was diagnosed with the ipsilateral left lung adenocarcinoma. (Video 1).

## CASE REPORT

**Figure 1:**
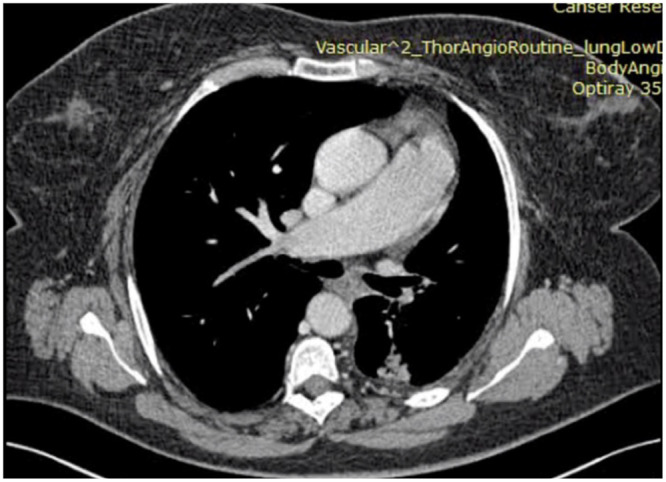
Preoperative computed tomography scan showed a unilateral absence of the left pulmonary artery.

**Figure 2:**
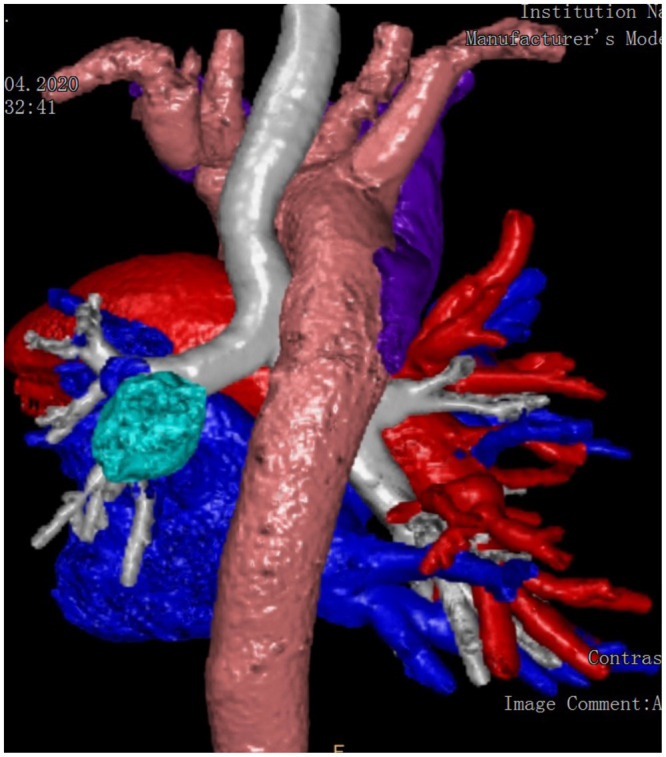
Three-dimensional reconstruction of the right-sided aortic arch and truncus pulmonalis, demonstrating the absence of the left pulmonary artery.

A 59-year-old female was admitted to our hospital. She presented with a mass in the left lower lobe detected on a chest X-ray during routine medical check-up. She had a 30-year smoking history and her past history include obesity with a body mass index of 40 kg/m^2^ and hypertension. Chest CT angiography showed a mass 2.6 × 2.3 cm in a superior segment of the left lower lobe and a hilum and mediastinal lymphadenopathy. Right-sided aortic arch and an absence of the left pulmonary artery were also found (Fig. 1). Also, computed tomography images revealed a hypoplastic left lung. Three-dimensional images of the chest anatomy were made and it also showed an absence of the left pulmonary artery and the presence of right-sided aortic arch (Fig. 2). There were no cardiac defects revealed on echocardiogram. Routine blood tests were within normal limits. A bronchoscopy demonstrated normal tracheobronchial anatomy of the right and left lungs. Pulmonary function tests showed a forced expiratory volume in 1 s of 1.95 l (79% predicted), forced vital capacity of 2.64 l (91% predicted) and a forced expiratory volume in 1 s to forced vital capacity ratio of 0.74. Transthoracic fine-needle biopsy of the tumour was performed and adenocarcinoma on biopsy samples was confirmed.

A primary diagnosis of left lung cancer cT1cN2M0 was made and 4 courses of the preoperative chemotherapy with paclitaxel 175 mg/m^2^ and carboplatin AUC 5 were performed. After preoperative chemotherapy, stabilization of the tumour process was declared and patient underwent fully thoracoscopic pneumonectomy with mediastinal lymphadenectomy. Intraoperatively, a mass up to 3.0 cm in diameter in the lower lobe was found. There were 2 pulmonary veins and no left pulmonary artery. Also, there were found 8 collateral vessels, also known as the left bronchial arteries that were dissected and clipped, made this operation difficult and unpredictable. Postoperative hospital stay was without any therapeutic and surgical complications. Discharge from the hospital was on postoperative Day 8. Histopathological examination confirmed adenocarcinoma of the left lung (ypT1pN2M0, уpIIIA stage).

## DISCUSSION

Unilateral absence of pulmonary artery is a very rare congenital anomaly, which is found in an isolated manner in up to 30% of patients. The most common clinical symptoms of patients with UAPA are dyspnoea, recurrent lung infections and haemoptysis. However, ∼15% of patients with UAPA have no symptoms and this anomaly is detected during routine check-up on X-ray or computed tomography scans. Unilateral absence of left pulmonary artery is commonly related with congenital heart defects, whereas right-sided UAPA is usually presented isolated [3]. The right-sided aortic arch is almost always associated with left pulmonary artery agenesis. In case of UAPA, affected lung receives blood supply from branches of the bronchial, phrenic, intercostal, subclavian or internal mammary arteries.

Approximately 440 cases of patients with unilateral absence of pulmonary artery are known up to date. Patients with UAPA associated with lung cancer are highly selective and rare. According to our data and search of the English literature, there are only 10 published cases and our case is the 11th one. Eight patients out of 10 had no symptoms, related to disease; 6 patients had unilateral absence of left pulmonary artery and 5 had it from the right side; ipsilateral side of UAPA was mentioned in 6 patients, whereas contralateral in 5. Location of the tumour was equally in the right and left lung; right-sided aortic arch was revealed in 3 cases; histopathologic diagnosis was adenocarcinoma in 7 patients, squamous cell carcinoma in 2, metastatic in 1 and not mentioned in 1. In all but one case surgical treatment was performed including lung resection, lobectomy or pneumonectomy. In 1 case, the diagnostic mediastinoscopy was done. To the best of our knowledge, the only minimally invasive surgery except limited lung resection in one case was performed in present case.

Currently, there is no clear algorithm of management and treatment of patients with UAPA accompanied by lung cancer. For patients with UAPA and ipsilateral lung cancer, surgical treatment would be a method of choice and pneumonectomy or lobectomy should be performed. Patients with UAPA and contralateral lung cancer belong to the more complex group. In this case, limited resections, such as segmentectomy or wedge resections, would be performed. Patients with a right pulmonary artery absence and left lung cancer are associated with a very high mortality risk. As an additional option before surgery embolization of collateral vessels can be performed to reduce intraoperative and postoperative bleeding.

## CONCLUSION

The present case describes a patient with adenocarcinoma of the left lower lobe, who was diagnosed left UAPA and right-sided aortic arch. This is the 11th reported case in the literature and the only one with performed fully thoracoscopic pneumonectomy. There is no direct evidence between development of lung cancer in patients with UAPA. Surgical treatment should be chosen based on the localization of lung cancer and the side of absence of pulmonary artery.

##  

**Conflict of interest:** none declared. 

### Reviewer information

Interactive CardioVascular and Thoracic Surgery thanks Amit Bhargava, Ilkka Ilonen and the other anonymous reviewers for their contribution to the peer review process of this article.
